# Effects of Prenatal Alcohol Exposure on Child Development

**Published:** 2002

**Authors:** Joseph L. Jacobson, Sandra W. Jacobson

**Affiliations:** Joseph L. Jacobson, Ph.D., is a professor in the Department of Psychology, College of Science, and Sandra W. Jacobson, Ph.D., is a professor in the Department of Psychiatry and Behavioral Neurosciences, School of Medicine, both at Wayne State University, Detroit, Michigan

Maternal alcohol use during pregnancy contributes to a range of effects in exposed children, including hyperactivity and attention problems, learning and memory deficits, and problems with social and emotional development.

## Fetal Alcohol Syndrome

The most serious consequence of maternal drinking during pregnancy is fetal alcohol syndrome (FAS). FAS was first described in the United States by Jones and Smith (1973), who identified a distinctive set of facial anomalies—short eyelid openings (palpebral fissures), flat midface, thin upper lip, and a flat or smooth groove between nose and upper lip (philtrum)—in children whose mothers drank very heavily during pregnancy. These children also exhibit growth retardation as well as significant cognitive and/or behavioral problems.

In contrast with Down syndrome patients, who exhibit impairment in virtually all aspects of intellectual function, FAS patients often perform relatively well on language tests (e.g., Kodituwakku et al. 1995), although they tend to have difficulty with complex language tests, especially those tapping the pragmatic aspect of language. The most consistent deficits are in arithmetic (Streissguth et al. 1991; Clarren et al. 1994) and attentional function (Kodituwakku et al. 1995). Although many FAS patients are mentally retarded (i.e., have an IQ less than 70), a substantial proportion perform in the low average to average range on IQ tests (Streissguth et al. 1991).

It is particularly instructive to consider studies comparing children with FAS with children not exposed to alcohol who have similar low IQ scores. In one such study, FAS children had reading scores similar to those of IQ-matched control subjects, but they performed significantly less well on arithmetic and certain aspects of attention (e.g., executive function, which is the ability to coordinate, plan, and execute appropriate responses and to modify behavior flexibly in response to feedback) (Carmichael Olson et al. 1998). Children with FAS have poorer socioemotional development (i.e., emotional, personality, social, and moral development) than would be expected based on their IQ scores (Thomas et al. 1998; Carmichael Olson et al. 1998).

## Fetal Alcohol Effects and Alcohol-Related Neurodevelopmental Disorder

The term “fetal alcohol effects” (FAE) is applied to children whose mothers are known to have drunk heavily during pregnancy and who exhibit some, but not all, of the characteristics of FAS (Streissguth et al. 1991; Coles et al. 1997). The IQ scores of FAE patients are also depressed but tend to be somewhat higher than those found in FAS children.

In an attempt to increase precision in diagnosis, an Institute of Medicine panel has recommended distinguishing among three forms of FAE (Stratton et al. 1996). The term “partial FAS” applies to children with confirmed heavy prenatal alcohol exposure, some components of the characteristic alcohol-related facial dysmorphology, and physical growth or neurodevelopmental abnormalities. “Alcohol-related birth defects” (ARBD) is applied to those with confirmed heavy prenatal alcohol exposure and one or more congenital abnormalities, usually cardiac, skeletal, renal, ocular, or auditory.

“Alcohol-related neurodevelopmental disorder” (ARND) is applied to children with confirmed heavy prenatal alcohol exposure who exhibit measurable, albeit generally subtler neurobehavioral deficits than are seen with FAS. Although reduced IQ scores are not usually found (Goldschmidt et al. 1996; Jacobson et al. 1998*a*; but see Streissguth et al. 1990), ARND children exhibit developmental deficits in the domains that are most severely affected by FAS. That is, the most consistent deficits are in arithmetic (Goldschmidt et al. 1996; Streissguth et al. 1993), attention (Streissguth et al. 1994; Jacobson et al. 1998*a*), and socioemotional function (Carmichael Olson et al. 1998; Jacobson et al. 1998*b*). In comparison with FAS, ARND affects a much larger number of children, but the effects, although clinically important, are less severe. Most recently, the term “fetal alcohol spectrum disorders” (FASD) has emerged to refer collectively to FAS, partial FAS, ARBD, and ARND.

The following sections examine in more detail the cognitive and behavioral effects of prenatal exposure to alcohol.

## Hyperactivity and Attention

Data on the relationship between FAS and hyperactivity are inconsistent. Although hyperactivity has been reported in several studies of clinic patients (Steinhausen et al. 1982; Nanson and Hiscock 1990), it was rated as least severe among the problems reported by parents of FAS children in a recent study (Roebuck et al. 1999). Coles and colleagues (1997) found little evidence of hyperactivity or impulsivity in a sample of FAS/FAE children recruited when their mothers sought prenatal care. These authors suggest that studies drawing participants from medical and psychiatric referrals, in contrast with longitudinal samples such as their own, may be more likely to include patients living in unstable family situations. As a result, the hyperactivity reported in studies of clinic-referred patients may have been caused by social and environmental factors, such as co-occurring attachment disorders, anxiety, and post-traumatic stress disorder. Clinic-referred samples may also be affected by selection bias. For example, FAS children who are also hyperactive are more likely to be referred for treatment because their behavior is disruptive in family and school settings.

### Sustained Attention

Sustained attention, which refers to the ability to remain alert and focused over time, is usually assessed by timed vigilance or continuous performance tests. In these tests, a series of letters is displayed on a computer screen and the child presses a button whenever a pre-designated target stimulus appears. Although Nanson and Hiscock (1990) found slower reaction time among FAS/FAE children on a vigilance task, the children’s error rates were not elevated, and other studies have not found deficits on vigilance tasks (Carmichael Olson et al. 1992; Coles et al. 1997). Sustained attention deficits become evident among FAS/FAE patients only on tasks that also require active processing of information. For example, Carmichael Olson and colleagues (1998) found poorer performance with increased prenatal alcohol exposure on a serial reaction time task, which requires remembering motor patterns of keystrokes on a computer, and on a timed reading comprehension test. As another example, Nanson and Hiscock (1990) found more errors among alcohol-exposed children than control subjects on a delayed reaction time test. These results demonstrate deficits primarily on sustained attention tasks that also require active recall of information or response inhibition, suggesting impairment in executive function rather than sustained attention per se.

### Focused Attention

At least three studies of FAS/FAE patients have reported deficits in focused attention, which is the ability to maintain attention in the presence of distraction (Carmichael Olson et al. 1992; Kerns et al. 1997; Coles et al. 1997). Poorer focused attention with increased prenatal alcohol exposure has also been found in children exposed primarily at the lower levels associated with ARND (Streissguth et al. 1994; Jacobson et al. 1998*a*). Coles and colleagues (1997) noted, however, that, although focused attention was significantly poorer among the FAS/FAE children than the control subjects in the study, the FAS/FAE children actually performed somewhat better than children with attention deficit hyperactivity disorder (ADHD). Thus, the focused attention deficit associated with prenatal alcohol exposure appears to be less severe than in ADHD.

### Cognitive Flexibility

Cognitive flexibility refers to the ability to attend to multiple criteria simultaneously and to shift attention during a task. FAS has been linked to poor cognitive flexibility on tests of verbal fluency in which the child is asked to list as many words as possible from a given category (Kodituwakku et al. 1995; Jacobson et al. 1998*a*). These tests assess the ability to monitor information retrieved from long-term memory for conformity with a prescribed rule (e.g., the given category). Reduced cognitive flexibility has also been found among FAS children on a design fluency test (Schonfeld et al. 2001), a visuospatial version of verbal fluency; the California Trail Making Test (Mattson et al. 1999), in which the child must alternate between successive numbers and letters while “connecting the dots”; and the Wisconsin Card Sorting Test (Kodituwakku et al. 1995; Coles et al. 1997). In the Wisconsin Card Sorting Test, the child must sort cards based on one of three underlying principles: color, shape, or number of items on a card. After the child utilizes the correct criterion for 10 successive trials, the criterion is changed. Inability to modify one’s responses when the criterion changes and perseveration on the wrong category indicate lack of flexibility. Thus, the test assesses both the ability to use feedback to alter one’s response and the ability to inhibit a previously learned but now inappropriate response (i.e., response inhibition). Poor response inhibition has also been found in FAS/FAE children on the California Stroop Test (Mattson et al. 1999), which measures speed in reading color names printed in a different color (e.g., the word “blue” printed in the color red) and on a modified design fluency task (Schonfeld et al. 2001).

### Planning

With regard to planning, FAS children show poor performance on tests such as the Stepping Stone Maze, Raven’s Standard Progressive Matrices, and two variants of the Tower of Hanoi: the Progressive Planning Test (Kodituwakku et al. 1995) and the Tower of California (Mattson et al. 1999). The Stepping Stone Maze assesses a child’s ability to use feedback to find an invisible path through a maze (Carmichael Olson et al. 1992); Raven’s Standard Progressive Matrices requires the child to determine which of six complex patterns is the most appropriate to insert in a blank space cut from a larger design; and the Tower of Hanoi involves moving beads on three colored pegs to match the pattern shown in a photograph. All three tasks assess complex planning, including the ability to analyze a problem, devise a strategy, monitor one’s performance, and modify one’s strategy as performance proceeds. Poorer executive function has also been found in studies that tested children exposed to alcohol primarily at levels associated with ARND. These studies used the Stepping Stone Maze (Streissguth et al. 1994) and a variant of the Tower of Hanoi (Jacobson et al. 1998*a*). Coles and colleagues (1997) noted that, in contrast with focused attention, executive function deficits were more severe in FAS/FAE children than in children with ADHD.

## Learning and Memory

Recent studies have found that FAS/FAE patients show greater impairment of certain aspects of learning and memory than others. Kerns and colleagues (1997) reported that, although nonretarded adults with FAS found it difficult to memorize word lists on the California Verbal Learning Test (CVLT), they had little apparent difficulty in retaining what they learned. Similarly, Mattson and colleagues (1996, 1998) found that FAS/FAE children tested on the CVLT have more difficulty in memorizing new information than in retaining and retrieving what they have previously learned.

In a study comparing FAS/FAE children with Down syndrome children, Mattson and Riley (1999) administered a priming task, in which the child initially reads a list of words and is then shown a list in which only the first two letters of the word are displayed. Some of the words on the second list come from the first list; others do not. Although both the alcohol-exposed and Down syndrome children performed more poorly than control subjects when asked to recall a list of words they had seen without any prompting or priming, the alcohol-exposed children performed as well as the control children in recognizing those words when presented in a multiple-choice format and when given the clues provided in the priming task. Thus, the learning and memory impairment associated with prenatal alcohol exposure is apparently more circumscribed than that associated with Down syndrome. Retention and recognition memory are relatively intact, as is the capacity to benefit from priming.

In another study, FAS/FAE children with normal range IQ scores were given 8 trials to learn to press 5 computer keys in a particular 10-item sequence (Carmichael Olson et al. 1998). The alcohol-exposed children were as capable of learning to perform the sequence manually (demonstrating procedural memory) as the controls but, when asked to verbally recall the sequence (demonstrating declarative memory), they were not able to do so. Thus, their procedural memory was apparently not affected.

Memory deficits have also been reported in children exposed at levels associated with ARND. Among 7-year-olds, greater prenatal alcohol exposure was associated with poorer memory for designs (Streissguth et al. 1989), poorer recall of number sequences (Streissguth et al. 1989; Jacobson et al. 1998*a*), and poorer recall of rhythmical patterns on the Seashore Rhythm Test (Streissguth et al. 1989).

In the only study to examine memory processing during infancy, Jacobson and colleagues (1993) found that, as in the studies of FAS/FAE children, recognition memory appeared to be unaffected by prenatal alcohol exposure. However, greater prenatal alcohol exposure was associated with slower, less efficient information processing at 6.5 and 12 months of age on two tasks involving the encoding of information into short-term memory (Jacobson et al. 1993). Greater prenatal alcohol exposure was also associated with the poorer performance on Visual Expectancy Paradigm (Haith et al. 1988), which assesses the degree to which the infant visually anticipates the next appearance of a stimulus during a regular left–right alternating display (Jacobson et al. 1994).

## Socioemotional Function

Prenatal alcohol exposure is associated with increased levels of irritability during infancy (Coles et al. 1991), a temperamental variable known to contribute to poorer maternal attachment and behavioral problems in childhood (Kelly et al. 2000). Two studies have found that children exposed prenatally to alcohol were rated by their teachers as less socially competent and more aggressive in the classroom (Brown et al. 1991; Jacobson et al. 1998*b*). Because these effects remained significant after controlling for current maternal drinking and measures of quality of parenting, these studies suggest that prenatal alcohol exposure may have effects on socioemotional development that are independent of the social environment in which the child is raised.

Carmichael Olson and colleagues (1992) administered the Vineland Adaptive Behavior Scale, a measure of social skills and emotional maturity, to the parents of FAS/FAE adolescents. The adolescents’ most substantial deficits, based on the parents’ responses, were in the socialization domain, which assesses interpersonal skills and the ability to conform to social conventions. The most salient problems were failure to consider the consequences of one’s actions, lack of responsiveness to social cues, and poor interpersonal relationships (Streissguth et al. 1991). Whereas the Vineland scores in two other domains— communication and daily living skills—were roughly commensurate with the children’s IQ scores, their interpersonal skills averaged 20 points lower than expected based on IQ (Carmichael Olson et al. 1992).

Thomas and colleagues (1998) compared 15 FAS/FAE children with 15 normal control subjects and 15 control children matched for verbal IQ. The Vineland scores of the alcohol-exposed children were significantly lower than those of the IQ-matched control subjects, especially in the interpersonal skills domain, providing additional evidence that the social judgment and relationship problems exhibited by these children are not simply consequences of their intellectual limitations. Thomas and colleagues (1998) also found that the discrepancy between the FAS/FAE children’s chronological age and age-equivalent Vineland score increased as the children grew older. This finding is consistent with both the report by Coles and colleagues (1991) of normal Vineland scores at age 6, when FAS children are frequently characterized as talkative, affectionate, and outgoing, and with the findings by Steinhausen and colleagues (1993) that behavior problems which become evident during childhood do not improve as the FAS patient reaches adulthood.

On the Personality Inventory for Children (PIC), the two domains identified by parents of school-age FAS/FAE children as most problematic were cognitive function and delinquency; the latter is not a prominent domain in most forms of mental retardation (Roebuck et al. 1999). These children were more likely to exhibit antisocial behaviors, lack consideration for the rights and feelings of others, and resist limits and requests by authority figures. This finding is consistent with the reports, cited above, of high levels of aggression in the classroom as well as a report by Streissguth and colleagues (1996) that adults with FAS are more likely to get into trouble with the law and to exhibit sexually inappropriate behavior.

## Caveats and Challenges

A detailed review of the current research on the developmental effects of prenatal alcohol exposure reveals some inconsistencies. For example, although arithmetic skill is frequently more impaired than verbal skills, some of the most severely affected patients perform poorly in both domains. Some of the inconsistencies could be caused by differences in the timing of exposure. Different brain regions and processes are most vulnerable at different points during gestation, but it is difficult to obtain accurate data regarding exactly when during the pregnancy the heaviest drinking occurred.

Other factors that lead to inconsistencies include limitations in the accuracy of reported quantities of alcohol ingested per occasion and individual differences in genetic vulnerability. Another limitation of the studies to date is that the domains assessed have been relatively global. Few studies have followed the example of Kopera-Frye and colleagues (1996), who evaluated specific aspects of arithmetic and found that cognitive estimation was more affected than computation per se.

The evidence linking prenatal alcohol exposure to deficits in socioemotional function is based on data from multiple sources, including ratings by parents and teachers and self-reports obtained from adolescents. However, there have been few direct observational studies to identify which specific aspects of socioemotional function are impaired (e.g., empathy, recognition of emotional expression, moral reasoning).

## Conclusion

In summary, these data indicate that prenatal alcohol exposure is associated with a distinctive pattern of intellectual deficits, particularly in arithmetic and certain aspects of attention, including planning, cognitive flexibility, and the utilization of feedback to modify a previously learned response. With respect to learning, the acquisition of new information is more likely to be impaired than retention and retrieval of previously learned information. As alcohol-exposed children grow older, deficits in socioemotional function become increasingly salient, particularly with regard to social judgment, interpersonal skills, and antisocial behavior. Although these deficits are most severe and have been documented most extensively in children with FAS, children prenatally exposed to lower levels of alcohol frequently exhibit similar problems.
